# *p63* silencing induces epigenetic modulation to enhance human cardiac fibroblast to cardiomyocyte-like differentiation

**DOI:** 10.1038/s41598-022-15559-y

**Published:** 2022-07-06

**Authors:** Jaya Pratap Pinnamaneni, Vivek P. Singh, Mary B. Kim, Christopher T. Ryan, Aarthi Pugazenthi, Deepthi Sanagasetti, Megumi Mathison, Jianchang Yang, Todd K. Rosengart

**Affiliations:** 1 Michael E. De Bakey Department of Surgery, Baylor College of Medicine, 1 Moursund St, Houston, TX-77030 USA; 2 Department of Surgery, Mount Sinai Hospital, New York, NY 10029 USA

**Keywords:** Cardiology, Cardiovascular biology, Cardiac regeneration, Gene therapy

## Abstract

Direct cell reprogramming represents a promising new myocardial regeneration strategy involving in situ transdifferentiation of cardiac fibroblasts into induced cardiomyocytes. Adult human cells are relatively resistant to reprogramming, however, likely because of epigenetic restraints on reprogramming gene activation. We hypothesized that modulation of the epigenetic regulator gene *p63* could improve the efficiency of human cell cardio-differentiation. qRT-PCR analysis demonstrated significantly increased expression of a panel of cardiomyocyte marker genes in neonatal rat and adult rat and human cardiac fibroblasts treated with *p63* shRNA (shp63) and the cardio-differentiation factors Hand2/Myocardin (H/M) versus treatment with Gata4, Mef2c and Tbx5 (GMT) with or without shp63 (p < 0.001). FACS analysis demonstrated that shp63+ H/M treatment of human cardiac fibroblasts significantly increased the percentage of cells expressing the cardiomyocyte marker cTnT compared to GMT treatment with or without shp63 (14.8% ± 1.4% versus 4.3% ± 1.1% and 3.1% ± 0.98%, respectively; p < 0.001). We further demonstrated that overexpression of the p63—transactivation inhibitory domain (TID) interferes with the physical interaction of p63 with the epigenetic regulator HDAC1 and that human cardiac fibroblasts treated with p63-TID+ H/M demonstrate increased cardiomyocyte marker gene expression compared to cells treated with shp63+ H/M (p < 0.05). Whereas human cardiac fibroblasts treated with GMT alone failed to contract in co-culture experiments, human cardiac fibroblasts treated with shp63+ HM or p63-TID+ H/M demonstrated calcium transients upon electrical stimulation and contractility synchronous with surrounding neonatal cardiomyocytes. These findings demonstrate that *p63* silencing provides enhanced rat and human cardiac fibroblast transdifferentiation into induced cardiomyocytes compared to a standard reprogramming strategy. p63-TID overexpression may be a useful reprogramming strategy for overcoming epigenetic barriers to human fibroblast cardio-differentiation.

## Introduction

Congestive heart failure typically occurring as a result of myocardial infarction remains the leading cause of mortality from cardiovascular disease^1–3^. Direct cellular reprogramming represents a novel strategy whereby resident cardiac fibroblasts in areas of myocardial infarction or fibrosis can be transdifferentiated into functional cardiomyocyte-like cells (iCMs) that can in turn enhance myocardial contractile function^4–7^. We and others have shown that different combinations of cardio-differentiating factors can transdifferentiate rodent and human cardiac fibroblasts into iCMs^8–14^. Human cells nevertheless appear resistant to reprogramming compared to rodent cells, likely due to epigenetic downregulation of cell plasticity^15–17^.

Given the effects of the *p53*-family of tumor suppressor genes in inhibiting pluripotent cell reprogramming^18–22^, we speculated that these genes could also impede the transdifferentiation of fibroblasts into cardiomyocytes by acting as ‘‘anti-plasticity” genes, especially in human cells^15–17^. Considering the known oncogenicity associated with *p53* downregulation, we elected to examine the potential role of *p63*, a *p53* family member with negligible known human oncogenicity^23–25^, as an anti-plasticity epigenetic regulator and *p63* silencing as a potential means to enhance human cell reprogramming. We accordingly showed that *p63*−/− mouse embryonic fibroblasts exhibit increased cardiomyocyte-like features compared to wild type cells^8^. We then demonstrated that *p63* silencing induced by administration of the short hairpin RNA for p63 (shp63) together with the cardiogenic differentiation factors Hand2 and Myocardin (H/M) also increases the expression of cardiomyocyte markers in mouse and human cardiac fibroblasts compared to their treatment with control vector^8^.

These findings led us to the current studies investigating whether epigenetic mechanisms were responsible for the effects of *p63* downregulation in enhancing rodent cell reprogramming, and if similar strategies could be used to enhance human cardiac fibroblast cardio-differentiation. In these studies, we demonstrated that overexpression of the p63—Transactivation inhibitory domain (TID), the p63 motif responsible for binding to the epigenetic regulator histone deacetylase 1 (HDAC1)^26^, was a potent replacement for shp63 in enhancing human cardio-differentiation.

## Methods

### Tissue collection and isolation of cardiac fibroblasts

Neonatal and adult cardiac fibroblasts were harvested using standard cell isolation techniques from 0–3 day-old to 6–8 week-old rats, respectively (Harlan Sprague Dawley Inc, Indianapolis, IN)^9,10,27^. All animal experiments were approved by Institutional Animal Care and Use Committee (IACUC) at Baylor College of Medicine and all methods were carried out in accordance with the NIH guidelines (Guide for the care and use of laboratory animals) and under protocol AN-6223. These studies were conducted and are reported in compliance with relevant elements of ARRIVE guidelines.

Adult human cardiac fibroblasts were isolated using standard isolation techniques from ventricular myocardial tissue obtained from explants of heart failure patients undergoing mechanical assist device placement or cardiac transplantation at Baylor St. Luke’s Medical Center^9,10^. A written informed consent was obtained from all the subjects and/or their legal guardian(s) prior to obtaining the tissue. All experimental methods were carried out in accordance with relevant guidelines and regulations under a protocol approved by the Baylor College of Medicine Institutional Review Board (IRB H‐33421). Briefly, explanted tissues were minced and then cultured in DMEM, 10% fetal bovine serum (FBS) and 1% penicillin/streptomycin. Fibroblasts were thereby allowed to migrate out from these explants over a period of 2 weeks, after which they were passaged three times in M106 medium (M106500; Thermo Fisher Scientific), 10% FBS, and LSGS kit supplements (S‐003‐K; Thermo Fisher Scientific).

### Cell reprogramming

Lentivirus vectors each encoding Gata4, Mef2, or Tbx5 (GMT), Hand2/Myocardin (H/M), non-targeting (NT) shRNA, *p63* short hairpin RNA (Origene, Rockville, MD), *p63*-transactivation inhibitory domain (Vectorbuilder, Chicago, IL) tagged with green fluorescent protein (GFP) or GFP control vectors were prepared from relevant plasmids by the Baylor College Of Medicine Gene Vector Core, as previously described^9,10,27,28^.

Rat and human cardiac fibroblasts isolated as described above were seeded onto 6 cm or 10 cm culture dishes for fluorescence‐activated cell sorting [FACS] analyses, onto 6‐well plates for quantitative reverse transcription polymerase chain reaction [qRT-PCR] analysis or onto 24‐well dishes pre-coated with Surecoat (SC-9035; Cellutron Life Technologies) for immunocytochemistry analyses. Twenty‐four hours after the cells were 70% to 80% confluent, lentiviral vectors at a multiplicity of infection (MOI) of 20 (unless otherwise indicated) were added to cell culture plates in a mixture with polybrene at a final concentration of 5 μg/μL. Two days after cell culture treatment with relevant reprogramming factors, the initial transfer medium (DMEM/199 [4:1], 10% FBS, and 1% penicillin/streptomycin) was replaced with induction medium (iCM media), as previously described. This media was replaced with fresh induction media every two days until cells were harvested^9,10^.

For cell contractility co-culture studies, cardiomyocytes were isolated from 0 to 3 day old rat pups under protocol AN-6223, as previously described^28–30^. Human cardiac fibroblasts were treated with GFP-labeled reprogramming factors (GMT, shp63+ H/M, p63-TID+ H/M) and one week after treatment as described above, cells were harvested and re-plated onto neonatal rat cardiomyocytes at a ratio of 1:10 and cultured in DMEM/M-199/10% FBS medium^31^.

### Flow cytometry

Fluorescence-activated cell sorting (FACS) was performed as previously described^8–10,27,28^. Briefly, cells adherent to culture dishes were first washed with DPBS and trypsinized with 0.25% trypsin/EDTA. Cells were then fixed with fixation buffer (BD Biosciences), washed with Perm/Wash buffer (BD Biosciences) and then incubated with mouse monoclonal anti-cardiac troponin T (cTnT) antibody (ab8295; Abcam) in Perm/Wash buffer. These cells were then incubated with donkey anti-mouse Alexa Fluor 647 (ab150107; Invitrogen™), washed 3× with Perm/Wash buffer again, and further analyzed for cTnT expression using a LSR Fortessa cell sorter (BD Biosciences) with FlowJo software (FlowJo, LLC, Ashland, Ore) and Diva software (version 6.0).

### Immunocytochemistry

Immunofluorescence (IF) staining was performed using cells fixed in 4% paraformaldehyde and permeabilized with 0.5% Triton‐X solution, as previously described^8–10,28^. After these cells were blocked with 10% goat serum, they were incubated with primary antibodies against cTnT (1:300 dilution; Thermo Fisher Scientific), or α-actinin (1:300 dilution; Sigma-Aldrich) followed by incubation with appropriate Alexa fluorogenic secondary antibodies (Invitrogen™). 4ʹ,6‐diamidino‐2‐phenylindole (DAPI; Invitrogen™) was used to stain nuclei. For quantification of cTnT and α-actinin positive cells, the ratio of cells expressing relevant IF markers versus total cells marked by DAPI was calculated in five random images selected by an investigator blinded to treatment group.

### qRT-PCR

Quantitative real-time polymerase chain reaction (qRT-PCR) analysis was performed by first extracting total RNA using the TRIzol method (Invitrogen™), as previously described^8–10,28,32^. Relative quantification of RNA was performed using SYBR green detection of PCR products in real time with the ABI ViiA 7 (Applied Biosystems Inc). Primers for qRT-PCR used in this study are listed in supplemental material (Supplemental Table S1). mRNA levels were normalized by comparative ΔΔCT method with comparison to glyceraldehyde 3-phosphate dehydrogenase (GAPDH).

### Co-immunoprecipitation (Co-IP) and western analyses

For Western analyses and Co-IP, 293T cells were transfected with plasmid vectors pcDNA3.1, ΔNp63α-FLAG (p63-FLAG; #26979, Addgene), HDAC1-GFP (#11054, Addgene) or p63-TID-HA (GenScript^®^) in lipofectamine™ 3000 Transfection Reagent (L3000008, Thermo Fisher Scientific). Cell lysates were collected and homogenized in cell lysis buffer. Protein was quantified using Pierce BCA protein assay kit (23227; Thermo Fisher Scientific) and Co-IP was performed with quantified protein using Immunoprecipitation Kit (10007D; Invitrogen™) following manufacturer’s protocol. As a final step, samples were loaded onto SDS-PAGE and after separation, the protein bands were transferred to nitrocellulose membrane (IB301001; Invitrogen™).

Immune detection was performed with the following primary antibodies: FLAG tag (F1804-200UG; Sigma-Aldrich), HDAC1 (sc-7872; Santa Cruz Biotechnology, Inc), β-Actin (sc-47778; Sigma-Aldrich), HA tag (sc-57592; Santa Cruz Biotechnology, Inc,), or TP63 (GTX 102425; GeneTex), followed by treatment with appropriate HRP–conjugated secondary antibodies (Millipore, Billerica, MA). Membranes were then washed with 1× Tris-buffered saline with Tween 20 and visualized by chemiluminescence detection (WBLUF0500; Millipore Sigma).

### Chromatin immunoprecipitation (ChIP)-qPCR assay

ChIP-qPCR assays were performed on murine embryonic fibroblasts isolated from *p63* flox/flox (f/f) mice (gift of Dr. Elsa Flores; Moffitt Cancer Center & Research Institute), as previously described^8^. Briefly, cells were seeded onto 150 mm dishes at density of 10^6^ cells/dish, and treated 24 h later with adenoviral vectors expressing GFP or Cre recombinase (AdGFP and AdCre; 150 moi). These cells were collected seven days later and processed using ChromaFlash Chromatin Extraction kits (p-2001; Epigenetek) per manufacturer’s protocol. ChIP was then conducted using H3K27Ac antibody (ab4729; Abcam), HDAC1 antibody (815104; Biolegend) or normal rabbit immunoglobulin G (AB-105-C; R&D systems), as previously described^27^. ChIP-qPCR was performed using primers synthesized by Sigma Aldrich (Supplemental Table S2). Fold-enrichment of PCR products was calculated after normalization with input of all three types of infected cells.

### Measurements of contractility and calcium transient

Cell contractility (cell shortening) and calcium transients in co-culture studies were measured at room temperature (22–23 °C). To perform these studies, cells were placed in plexiglass chamber which was positioned on the stage of an inverted epifluorescence microscope (Nikon Diaphot 2000) and perfused with 1.8 mmol/L Ca^2+^‐Tyrode’s solution containing (in mmol/L): NaCl 140, KCl 5.4, MgCl_2_ 1, CaCl_2_ 1.8, HEPES 5, and glucose 10, pH 7.4. Cells that had been previously treated with reprogramming factors were identified by GFP fluorescence.

Field-stimulation was provided by a Grass S5 stimulator using platinum electrodes placed alongside a cell culture bath containing 1.8 mM Ca^2+^, with bipolar pulses delivered at voltages 50% above myocyte stimulation thresholds. Contractions of iCMs from random fields were videotaped and digitized on a computer. For Ca^2+^ signal measurements, cells were loaded with 3 μmol/L of Fura‐2/AM (Life Technologies) and alternately excited at 340 and 380 nm at 0.5 Hz by use of a Delta Scan dual‐beam spectrophotofluorometer (Photon Technology International, Edison, NJ). Ca^2+^ transients were expressed as the 340/380‐nm ratios of the resulting 510‐nm emissions. Data were analyzed using Felix software (Photon Technology International)^8,9,28–30^.

### Statistical analyses

Three independent biological replicates each measured in technical triplicates were performed for all studies. All data are expressed as the mean ± standard error (SEM). Statistical analysis was performed using SAS, version 9.4. Student’s *t*-test was used to determine significance of differences between two groups. One-way ANOVA was used to determine the significance of differences when more than 2 groups were compared.

## Results

### *p63* silencing promotes rat cardiac fibroblast cardio-differentiation

**Figure 1:**
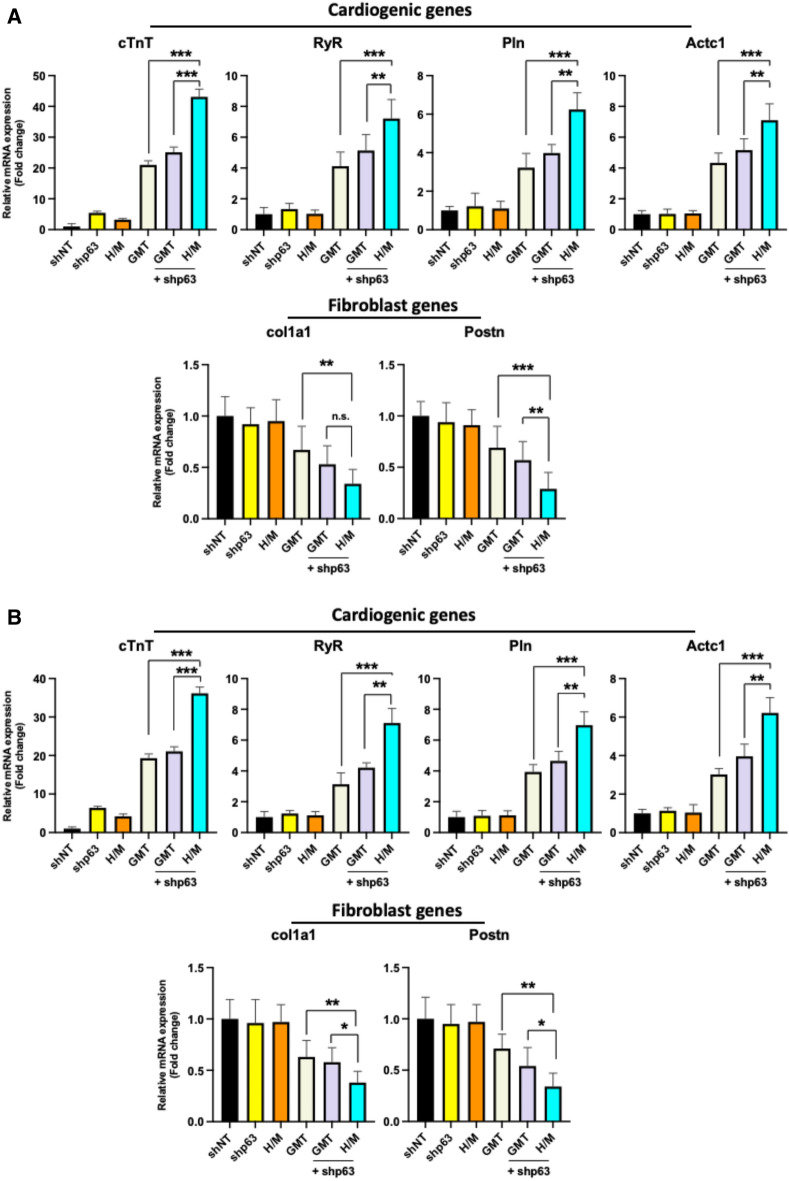
*p63* silencing in combination with Hand2/Myocardin enhances cellular reprogramming in rat cardiac fibroblasts. Cardiomyocyte and fibroblast marker gene expression in neonatal (**A**) and adult (**B**) rat cardiac fibroblasts (CFs), as assessed by qRT-PCR two weeks after treatment with shp63 with or without Hand2/Myocardin (H/M) versus treatment with Gata4, Mef2c, and Tbx5 (GMT) with or without shp63 (n = 3, ***p < 0.001, **p < 0.01, *p < 0.05, *n.s.* not significant), *shNT* short hairpin non-targeting.

Significantly increased expression of a panel of cardiogenic marker genes (*cTnT, RyR, Pln, Actc1*) was demonstrated by qRT-PCR analysis of neonatal and adult rat cardiac fibroblasts treated with shp63 together with Hand2/Myocardin (H/M) compared to cells treated with a standard GMT reprogramming cocktail with or without shp63 (p < 0.001; Fig. 1A,B). In comparison, cardiogenic gene expression was unchanged in cells treated with either shp63 or H/M alone versus cells treated with non-targeting shRNA. Likewise, cells treated with shp63 together with H/M exhibited decreased expression of fibroblast marker genes (*col1a1, Postn*) compared to cells treated with GMT with or without shp63 (p < 0.01; Fig. 1A,B; Supplemental Fig. S1).

### *p63* silencing significantly enhances human cardiac fibroblast cardio-differentiation

**Figure 2:**
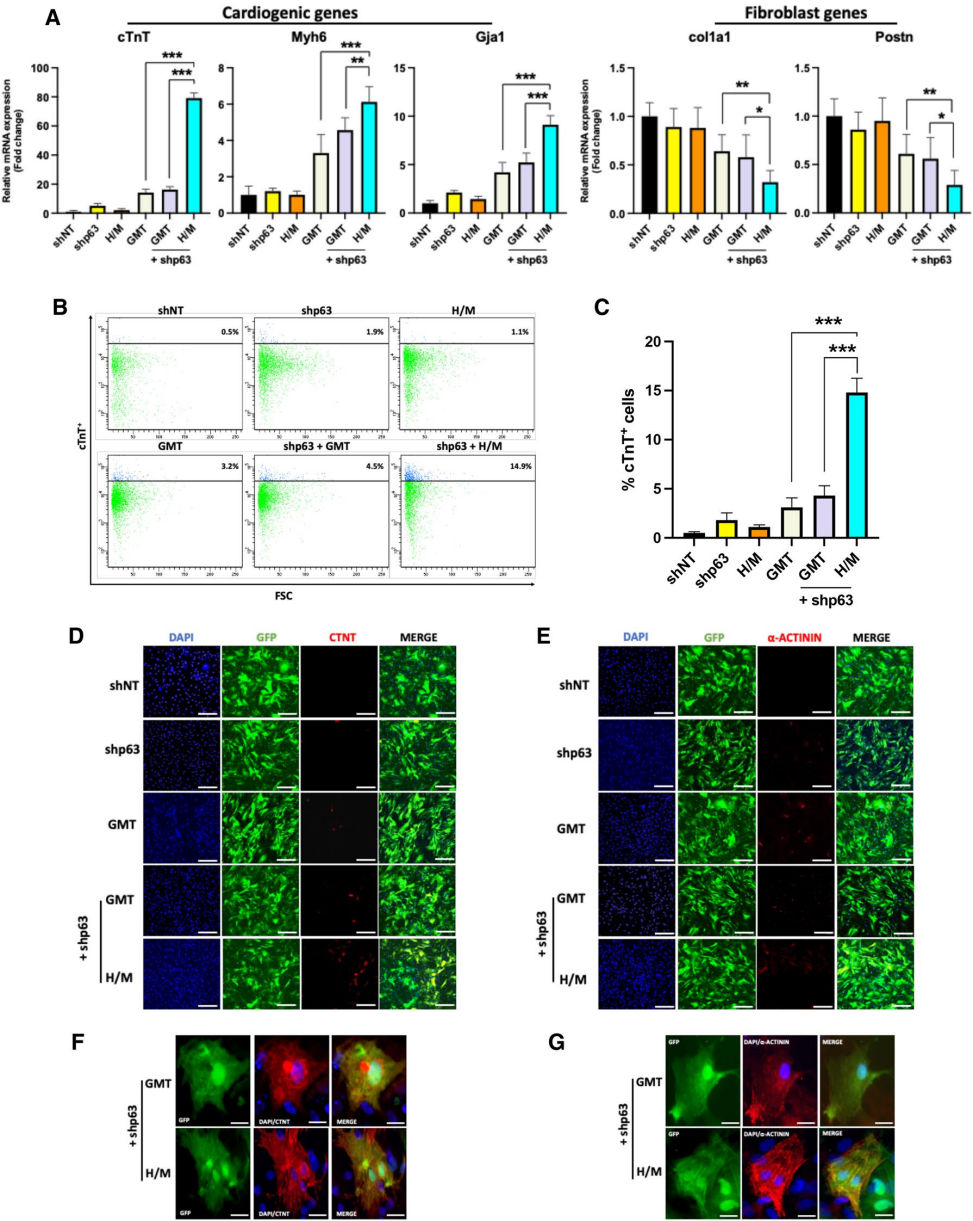
*p63* silencing in combination with Hand2/Myocardin enhances human cardiac reprogramming. (**A**) Cardiomyocyte and fibroblast marker gene expression assessed by qRT‐PCR (n = 3, ***p < 0.001, **p < 0.01, *p < 0.05), *shNT* short hairpin non-targeting. (**B**) Representative flow cytometry plots for cardiac troponin T positive (cTnT ^+^) human cardiac fibroblasts 2 weeks after their treatment with shp63 with or without Hand2/Myocardin (H/M) versus GMT) with or without shp63. (**C**) Quantification of the percentage of cTnT^+^ cells treated as indicated, as assessed by flow cytometry (n = 3; ***p < 0.001). (**D,E**) Representative immunofluorescence staining for 4ʹ,6‐diamidino‐2‐phenylindole (DAPI) (blue), GFP (green), and (red) cardiomyocyte markers cTnT (**D**) and α‐actinin (**E**) after 2 weeks. Scale bar = 100 μm. (**F,G**) Representative high magnification images of immunofluorescence staining for cTnT (**F**) and α-actinin (**G**) after 4 weeks of shp63+ H/M transduction demonstrating sarcomeric structures, most clearly visible in α-actinin labeled cells. Scale bar: 25 μm.

qRT-PCR analysis demonstrated that the expression of a panel of cardiomyocyte marker genes (*cTnT, Myh6 and Gja1*) was increased in human cardiac fibroblasts treated with shp63 and H/M compared to cells treated with GMT with or without shp63 group (p < 0.001; Fig. 2A). Analogous decreases in the expression of fibroblast marker genes (*col1a1, Postn*) were also observed in shp63+ H/M treated cells (p < 0.01; Fig. 2A). As with our observations of rat cardiac cells, administration of either shp63 or H/M alone failed to significantly alter cardiogenic or fibrogenic gene expression in human cardiac fibroblasts.

FACS analysis similarly demonstrated that the percentage of human cardiac fibroblasts expressing cTnT was also increased after treatment with shp63+ H/M compared to cells treated with GMT with or without shp63 (14.8% ± 1.4% versus 4.3% ± 1.1% and 3.1% ± 0.98%, respectively; p < 0.001; Fig. 2B,C). Immunofluorescence analyses likewise demonstrated a greater number of cells expressing the cardiomyocyte markers cTnT and α‐actinin 2 weeks after treatment with shp63+ H/M versus GMT with or without shp63, but neither treatment group exhibited clear sarcomeric ultrastructure (Fig. 2D,E). Four weeks after reprogramming factor treatment, fourfold more cells treated with shp63+ H/M exhibited α-sarcomeric actinin^+^ expression and shp63+ H/M treated cells exhibited advanced sarcomere organization compared to cells treated with shp63 and GMT (Fig. 2F,G).

### p63 recruits HDAC1 to modulate reprogramming of cardiogenic genes

**Figure 3:**
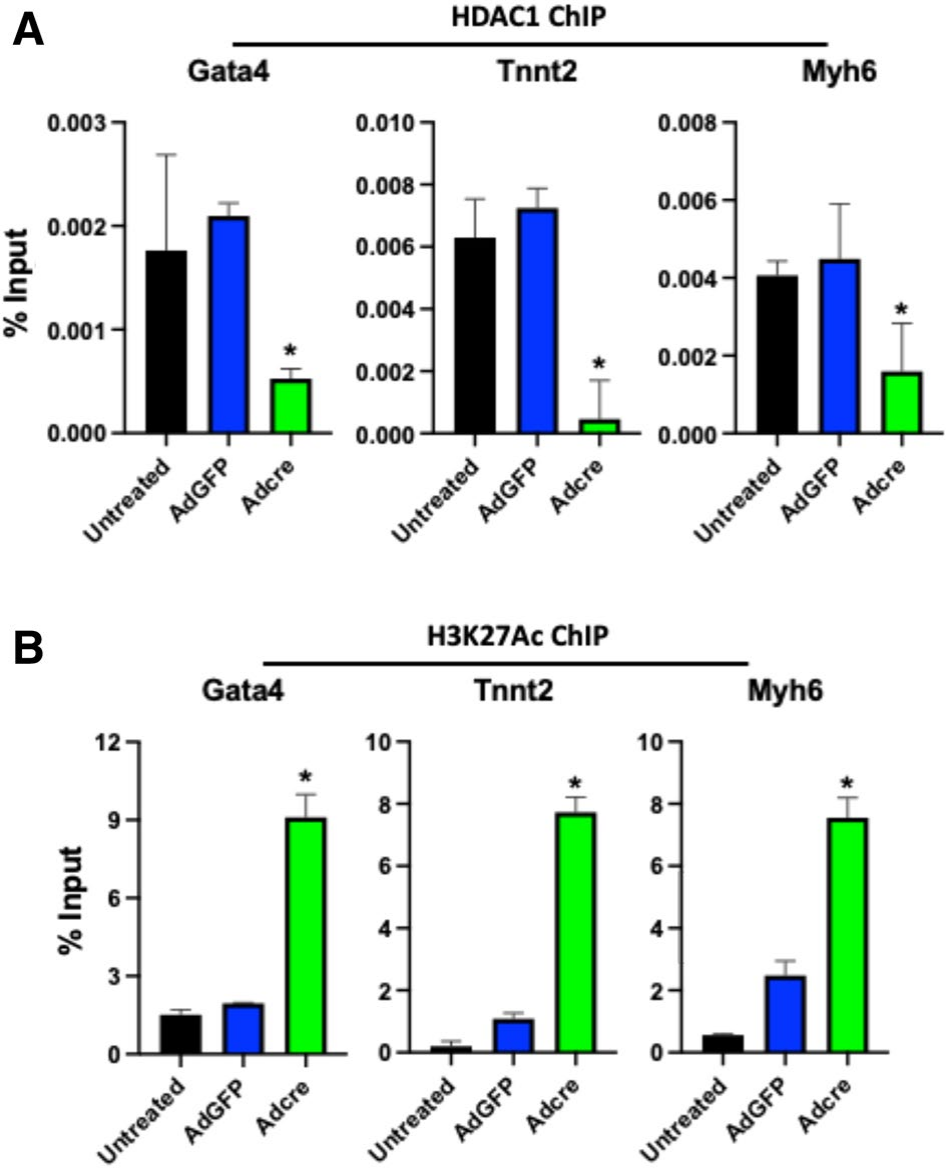
p63 recruits HDAC1 at cardiogenic gene promoter regions. Enrichment ChIP analysis of HDAC1 (**A**) and H3K27Ac (**B**) levels in promoter regions of cardiogenic genes indicated in AdGFP (control) or AdCre (*p63*^−/−^) treated *p63* flox/flox murine embryonic fibroblasts after 7 days (n = 3; *p < 0.01 versus AdGFP).

Co-IP and Western analyses demonstrated that p63 physically interacts with the epigenetic regulator HDAC1 (Supplemental Fig. S2). Furthermore, ChIP-qPCR analysis demonstrated decreases in HDAC1 levels at the promoter sites of cardio-differentiation genes (*Gata4, Tnnt2, Myh6*) one week after *p63* flox/flox murine embryonic fibroblasts were treated with AdCre (*p63*^−/−^) compared to cells treated with AdGFP (Fig. 3A, p < 0.01). Moreover, H3K27Ac ChIP revealed upregulation of this active enhancer mark at these gene promoter sites (Fig. 3B, p < 0.01) (Supplemental Fig. S3).

### p63-TID substitutes for shp63 in enhancing human cell cardio-differentiation

**Figure 4:**
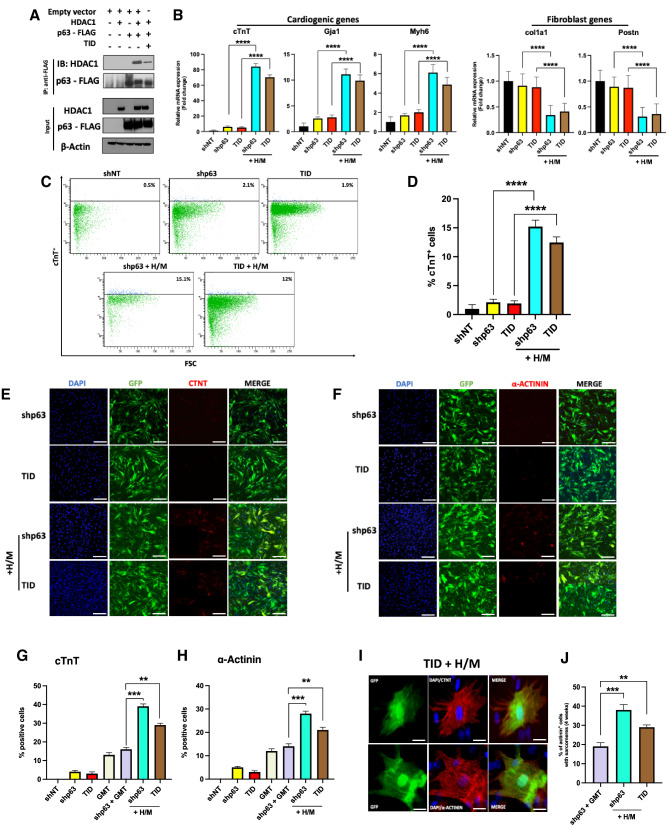
p63-TID interferes with p63/HDAC1 interactions and enhances human cardiac reprogramming. (**A**) FLAG co-immunoprecipitation assay in 293T cells transfected with HDAC1, p63-FLAG and/or p63-TID vectors, showing TID interference in p63-HDAC1 binding. Beta-actin was used as loading control. *IB* immunoblot, *IP* immunoprecipitation. (**B**) Cardiomyocyte and fibroblast marker gene expression assessed by qRT‐PCR after indicated treatments (n = 3, ****p < 0.0001). (**C**) Representative flow cytometry plots for cardiac troponin T positive (cTnT ^+^) human cardiac fibroblasts 2 weeks after their treatment with shp63 or p63-TID with or without Hand2/Myocardin (H/M). (**D**) Quantification of the percentage of cTnT^+^ cells treated as indicated, as assessed by flow cytometry (n = 3; ****p < 0.0001). (**E,F**) Representative immunofluorescence staining for DAPI (blue), GFP (green), and cardiomyocyte markers (red) cTnT (**E**) or α‐actinin (**F**) after 2 weeks. Scale bar = 100 μm. (**G**,**H**) Quantification of cells expressing cardiomyocyte markers cTnT^+^ and α‐actinin^+^ 2 weeks after indicated treatments, as assessed by immunofluorescence labelling (n = 3, ***p < 0.001, **p < 0.01). (**I**) Representative high‐magnification images of cTnT and α‐actinin staining in cells treated with p63-TID and H/M demonstrating sarcomeric structures, most clearly visible in α-actinin labeled cells. Scale bar = 25 μm. (**J**) Quantification of cells with well-developed sarcomeres as a percentage of total α-actinin^+^ cells after 4 weeks of shp63+ GMT, shp63+ H/M, or p63-TID+ H/M transduction (n = 3; **p < 0.01, ***p < 0.001).

Based on observations that the p63-transactivation inhibitory domain (TID) is crucial for the physical interaction of p63 with HDAC1^26^, we speculated that overexpression of p63-TID would block p63-mediated binding of HDAC1 to cardio-differentiation gene promoter sites. We were accordingly able to use Co-IP to confirm the competitive binding of p63-TID to HDAC1 (Fig. 4A; Supplemental Fig. S4). Based on these findings, we hypothesized that p63-TID could substitute for *p63* shRNA in enhancing human cardio-differentiation. While p63-TID treatment alone failed to alter cardiogenic or fibrogenic gene expression in human cardiac fibroblasts, qRT-PCR analysis demonstrated increased expression of a panel of cardiomyocyte marker genes (*cTnT, Gja1, Myh6*) by human cardiac fibroblasts treated with p63-TID+ H/M similar to that induced by shp63+ H/M (Fig. 4B). Cells treated with p63-TID+ H/M likewise displayed reduced expression of fibroblast marker genes (*col1a1, Postn*) comparable to that achieved with shp63+ H/M treatment (Fig. 4B).

FACS analysis similarly demonstrated that the percentage of human cardiac fibroblasts expressing cTnT was similarly increased after treatment with p63-TID+ H/M compared to shp63+ H/M treatment (12.5% ± 0.9% and 15.2% ± 1.1%, Fig. 4C,D). Immunofluorescence studies also demonstrated a similar threefold increase in the number of cells expressing the cardiomyocyte markers cTnT and α‐ actinin after treatment with p63-TID+ H/M or shp63+ H/M versus cells treated with shp63+ GMT (p < 0.001; Fig. 4E–H). Four weeks after reprogramming factor treatment, threefold more cells treated with p63-TID+ H/M exhibited α-sarcomeric actinin^+^ expression and p63-TID+ H/M treated cells exhibited advanced sarcomere organization compared to cells treated with shp63 and GMT (Fig. 4I,J).

### p63 silencing induces iCM contractility

**Figure 5:**
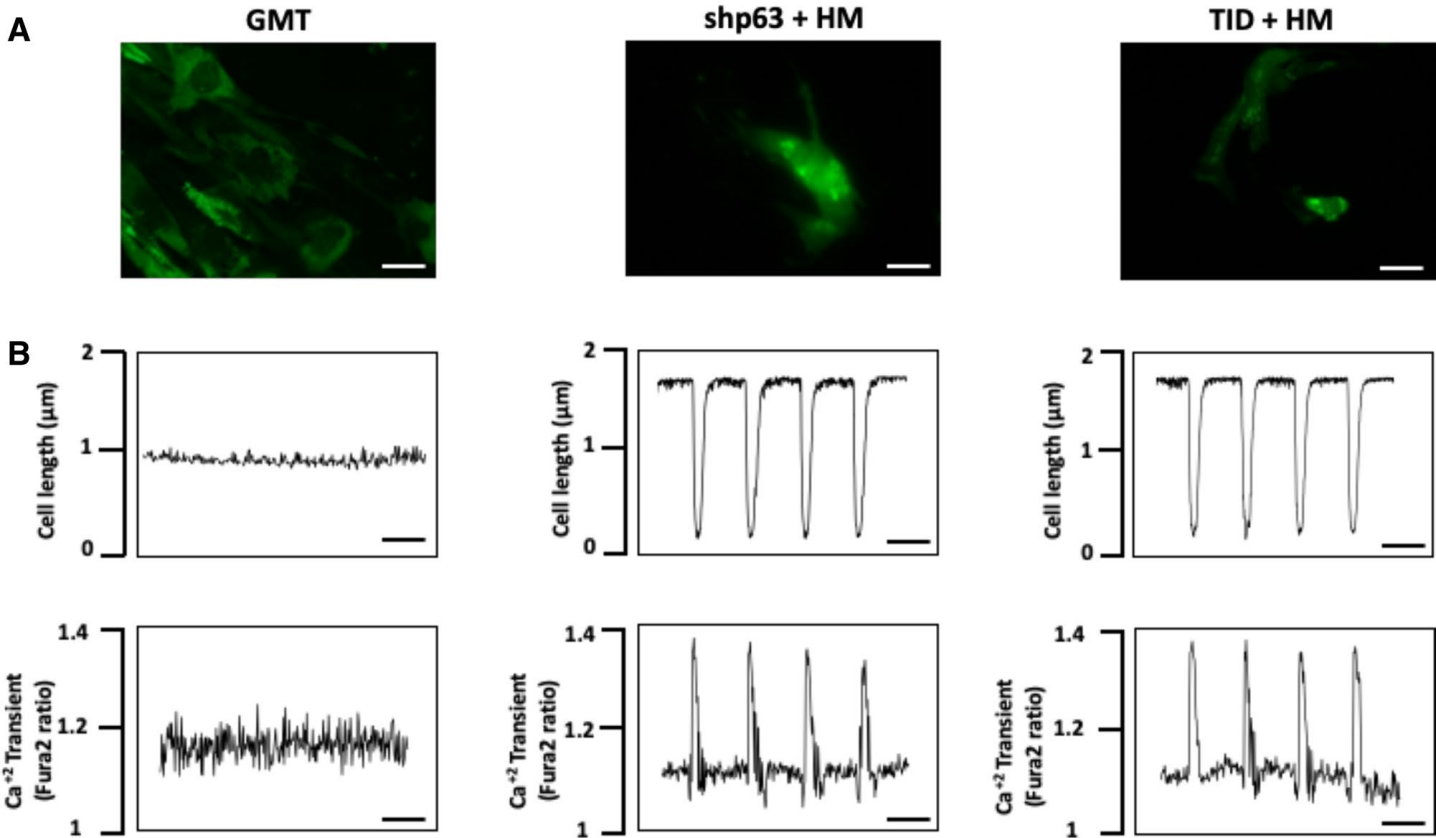
Functional efficacy of human cardiac fibroblast reprograming after co-culture with neonatal rat cardiomyocytes. Adult human cardiac fibroblasts were treated with lentivirus expressing GMT (left), shp63 in combination with Hand2/Myocardin (middle) or p63-TID+ Hand2/Myocardin (right). One week after initial transduction, these human cardiac fibroblasts were co-cultured with (untreated) neonatal rat cardiomyocytes (negative for GFP [green fluorescent protein]). (**A**) Representative immunofluorescence demonstrating (green) GFP expression by human cardiac fibroblasts treated with GMT (left), shp63+ H/M (middle) or p63-TID+ H/M (right) after 4 weeks in co-culture with (non-transduced) neonatal rat cardiomyocytes. Bar = 100 μm. (**B**) Representative peaks from GFP^+^ human cardiac fibroblasts treated with GMT, shp63+ H/M and p63-TID+ H/M after 4 weeks of co-culture, reflecting contraction (top row) and Ca^2+^ transients (bottom row). Contractility parameters were not observed in cells treated with GMT alone. Bar = 1 s.

Although human cardiac fibroblasts treated with shp63+ H/M or p63-TID+ H/M were not observed to contract independently, approximately ≈ 5% of human cardiac fibroblasts treated with shp63+ H/M or TID+ H/M, as verified by their GFP expression, contracted synchronously with surrounding neonatal rat cardiomyocytes after 4 weeks in co-culture (Fig. 5A). In comparison, human cardiac fibroblasts treated with GMT with or without shp63 failed to contract in co-culture experiments (Supplemental Videos S1, S2, S3). Cells treated with shp63+ H/M or p63-TID+ H/M also demonstrated calcium transients upon electrical stimulation that was synchronous with their contractile function, whereas calcium transients were not observed after stimulation of cells treated with GMT with or without or shp63 (Fig. 5B, Supplemental Video S1, S2, S3).

### p63-TID dose response and enhanced potency versus shp63 in enhancing human cardio-differentiation

**Figure 6:**
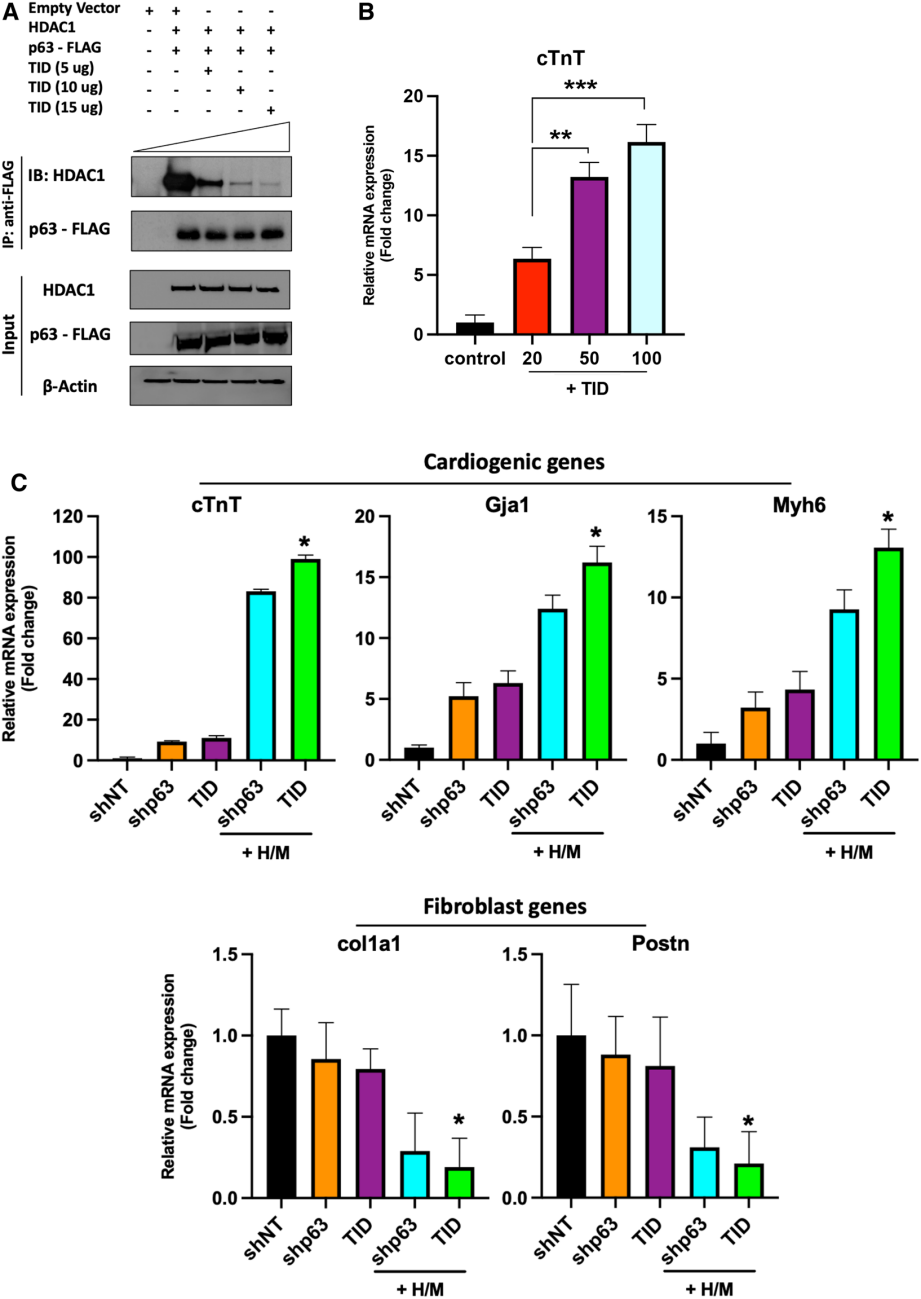
Dose-based efficacy of p63-TID vs shp63 in enhancing human cardiac reprogramming. (**A**) FLAG co-immunoprecipitation assay in 293T cells transfected with HDAC1, p63-FLAG and/or p63-TID vectors at three different p63-TID dosages showing increasing interference in p63-HDAC1 binding as a function of p63-TID dosage. Beta-actin was used as loading control. *IB* immunoblot, *IP* immunoprecipitation. (**B**) Dosage screening of p63-TID in human cardiac fibroblasts, qRT-PCR analysis of cardiac Troponin T (cTnT) marker 2 weeks after human cardiac fibroblasts were treated with 20, 50 or100 MOI of lentiviral vector expressing p63-TID (n = 3; ***p < 0.001, **p < 0.01). Control: lentiviral GFP vector, 20 MOI. (**C**) Cardiomyocyte and fibroblast marker gene expression in human cardiac fibroblasts as assessed by qRT-PCR two weeks after indicated treatments using p63-TID vector at a multiplicity of infection (MOI) of 50 (n = 3, H/M = 20 MOI; *p < 0.05 versus shp63+ H/M).

To determine whether p63-TID was more potent than shp63 in enhancing cardio-differentiation, we used Co-IP analysis to generate a dose–response analysis of p63 binding to HDAC1 as a function of p63-TID overexpression (Fig. 6A; Supplemental Fig. S5). qRT-PCR analysis of human cardiac fibroblasts treated with p63-TID at an MOI of 20, 50 or 100 MOI demonstrated increased cTnT expression in a dose-dependent fashion, with an MOI of 50 providing the highest cTnT expression without cell toxicity (p < 0.001; Fig. 6B). We were accordingly able to use qRT-PCR of human cardiac fibroblasts treated at an MOI of 50 to demonstrate significantly greater changes in cardiogenic and fibrogenic gene expression after p63-TID+ H/M versus shp63+ H/M treatment (p < 0.05, Fig. 6C).

## Discussion

Direct cardiac reprogramming represents a novel strategy whereby resident cardiac fibroblasts in areas of myocardial infarction or fibrosis can be transdifferentiated into functional cardiomyocyte-like cells that can in turn enhance myocardial contractile function^1–3^. We and others have previously reported that the administration of various combinations of cardio-differentiation transcription factors and chemical compounds can effectively induce the reprogramming of cardiac fibroblasts into functional cardiomyocyte-like cells termed induced cardiomyocytes (iCMs)^8–10,27–29,32,33^. The administration of these factors alone appears insufficient, however, to effectively induce iCM generation from human fibroblasts, likely due to “anti-plasticity” epigenetic repression mechanisms that inhibit gene activation to an extent that appears greater in higher level species such as humans^9,10^.

In the current study, we show that rodent as well as human cardiac fibroblasts can be converted into contractile iCMs through a reprogramming strategy mediated by the silencing of the epigenetic effects of *p63*. Specifically, we demonstrate that shp63 in combination with the cardio-differentiation factors Hand2 and Myocardin (H/M) led to enhanced neonatal, adult rat and adult human cardiac fibroblast differentiation compared to their treatment with a standard reprogramming cocktail (i.e., Gata4, Mef2c and Tbx5 [GMT]) alone^5,10,34^. In comparison, neither shp63 nor H/M alone exerted significant reprogramming effects.

Our focus on *p63* in these studies stems from observations that the *p53* family of epigenetic regulator proteins plays an important role in impeding induced pluripotent stem cell (iPSC) reprogramming^18–20,23,35,36^. We speculated that silencing of *p63*, which appears to play a role similar to *p53* in repressing iPSC reprogramming without its oncogenic effects, might be an ideal reprogramming agent enhancing cardiac cell transdifferentiation and iCM generation^24,37–43^. Our present study confirms this hypothesis and specifically identifies p63 interactions with the epigenetic repressor HDAC1 as a potential mechanism of action underlying this effect. Our finding that p63 interacts with HDAC1 to initiate epigenetic re-patterning and modulate cardio-differentiation gene promoters confirms the role of epigenetic modulation as an important regulator of human cell cardiac reprogramming^44,45^.

The C-terminus of both of the two major isoforms of *p63* (TAp63, ΔNp63) contains a transactivation inhibitory domain (TID) that has been reported to play an important role in gene regulation via its interactions with HDAC1^26^. We consequently speculated that overexpression of TID could substitute for the use of shRNA to inhibit the epigenetic effects of p63 and inhibition of cell reprogramming. This study demonstrated that p63-TID could be used in this manner to enhance cardiogenic reprogramming gene activation.

The potency of our *p63* silencing strategy in inducing contractile iCMs compared to the use of a standard reprogramming cocktail could be related to its observed effects in influencing the regulation of a diverse panel of relevant cardiogenic and fibrogenic genes. In comparison, standard reprogramming cocktails have required the administration of each of these constituent reprogramming factors in order to achieve efficacy^11,31,34,46–48^. In this context, our addition of H/M as a supplement to *p63* silencing likely relates to status of H/M as the “missing element” complementing key cardio-differentiation factors such as GMT that are otherwise upregulated by our *p63* silencing strategy. It is likewise interesting that *p63* silencing leads to the downregulation of fibrogenic genes known to impede cardio-differentiation, which has likewise been addressed by others through the addition of anti-fibrogenic factors to reprogramming cocktails^9,10,46–48^.

In contrast to the potential advantages of our HDAC-directed reprogramming strategy, it is possible that use of this potentially non-specific strategy could lead to promiscuous activation or silencing of genes unrelated to desired cardio-differentiation effects, including oncogenes. This possibility is refuted, however, by the lack of such observations in prior reprogramming studies intervening on HDAC1 regulation^14,19^. Our prior studies likewise suggest that *p63* silencing is itself not oncogenic^8^. Nevertheless, the potential risks of promiscuous gene activation could be addressed by gene-directed modifications of our *p63* silencing strategy, including CRISPR-dcas9 based delivery^49–51^. These could be applied if needed based on our planned RNA-Seq studies seeking to identify such potentially deleterious gene expression profiles.

Taken together, the present study suggests that *p63* acts as an epigenetic barrier in human cardiac reprogramming and that p63-TID offers a new potential strategy to target epigenetic regulation of cardiogenic gene activation as a means to enhance human cardiac reprogramming.

### Study limitations

Our premise that shp63 knockdown in combination with Hand2/Myocardin leads to enhanced human cardiac reprogramming might involve signaling pathways and epigenetic mechanisms other than those we reported herein. We intend to perform additional analyses including RNAseq, ATACseq and ChIPseq studies to further investigate the existence of such alternative pathways. Additional studies are also underway to characterize and determine the optimum dosage for TID. Further studies are likewise needed to clarify the relationship between the role of TID in transcriptional activation of cardiac genes and epigenetic re-patterning during iCM reprogramming.

## Supplementary Information

Supplementary Information.

Supplementary Video S1.

Supplementary Video S2.

Supplementary Video S3.
