# Supplementary material for: p63 silencing induces epigenetic modulation to enhance human cardiac fibroblast to cardiomyocyte-like differentiation
Source: Sci Rep. 2022 Jul 6;12:11416. doi: 10.1038/s41598-022-15559-y (PMC9259667; doi:10.1038/s41598-022-15559-y)
Supplement: Supplementary file 1 — Supplementary Information. [file 41598_2022_15559_MOESM1_ESM.pdf]

SUPPLEMENTARY FIGURES

Title: ***p63* Silencing Induces Epigenetic Modulation to Enhance Human Cardiac Fibroblast to Cardiomyocyte-Like Differentiation**

Jaya Pratap Pinnamaneni, MS; Vivek P. Singh, PhD; Mary B. Kim, MD; Christopher T. Ryan; MD; Aarthi Pugazenthi, MS; Deepthi Sanagasetti, MS; Megumi Mathison, MD, PhD; Jianchang Yang, MD, PhD; *Todd K. Rosengart, MD.

From the Department of Surgery, Baylor College of Medicine, Houston, TX 77030, USA (J.P.P., V.P.S., J.Y., C.T.R., A.P., D.S., M.M., T.K.R.); From the Department of Surgery, Mount Sinai Hospital, NY, 10029, USA (M.B.K.)

*Correspondence to:

Todd K. Rosengart, MD

Michael E. De Bakey Department of Surgery

Baylor College of Medicine

1 Baylor Plaza, MS 390, Houston, TX-77030, USA.

Phone: 713-798-3020

Fax:713-798-6374

Email: todd.rosengart@bcm.edu

Supplemental Table S1. qRT-PCR rat and human primers list

Rat Primer sequences:

Gene	Forward Primer	Reverse Primer
cTnT	AGGCTCACTTCGAGAACAGG	ATTGCGAATACGCTGCTGT
Ryr	ACATCATGTTTTACCGCCTGAG	TTTGTGGTTATTGAACTCTGGCT
Pln	GTGACGATCACAGAAGCCAAGG	TGACAGCAGGCAGCCAAACG
Actc1	GATTATTGCTCCCCCTGAGCG	GTGTAAGGTAGCCGCCTCAGAA
Col1a1	GCGAAGGCAACAGTCGATTC	CCCAAGTTCCGGTGTGACTC
Postn	GGCTGAAGACTGCCTTGAATGAC	CGTGGCAGCACCTTCAAAGA
Gapdh	GGCACAGTCAAGGCTGAGAATG	ATGGTGGTGAAGACGCCAGTA
ΔNp63	GGAAAACAATGCCCAGACTC	GTGGAATACGTCCAGGTGGC
TAp63	AAGATGGTGCGACAAACAAG	AGAGAGCATCGAAGGTGGAG

Human Primer sequences:

Gene	Forward Primer	Reverse Primer
cTnT	AGACGCCTCCAGGATCTGT	TCTTCAACAGCTGCTTCTTCC
Myh6	GCTGGTCACCAACAATCCCTA	CGTCAAAGGCACTATCGGTGG
Gja1	GCCAAAGACTGTGGGTCTCA	GAAGGTCGCTGGTCCACAAT
Col1a1	CATGTTCAGCTTTGTGGACCT	GCAGCTGACTTCAGGGATGT
Postn	CTCATAGTCGTATCAGGGGTCG	ACACAGTCGTTTCTGTCCAC
Gapdh	TCACCACCATGGAGAAGGC	GCTAAGCAGTTGGTGGTGCA
ΔNp63	GAAAACAATGCCCAGACTCAA	TGCGCGTGGTCTGTGTTA
TAp63	TGTATCCGCATGCAGGACT	CTGTGTTATAGGGACTGGTGGAC

Table S2. ChIP-qPCR Primer list:

Gene	Forward Primer	Reverse Primer
Gata4_G1	CCAATCCTTCCTCTCTGAACC	TCAAGTGTCCGTTGATCTTCA
Gata4_G2	TGAAGCTCGGGGCTTAAGTG	AGCGAAGGATCACCCGATTC
Gata4_G3	CAGTTCCAGGGCGTCCAATA	TCTTCTGCGGGCATGCTAAT
Tnnt2_T1	GGCAGTGGAGGGTGTAACTG	CTCCCACCACCAACTGTCTG
Tnnt2_T2	TCAAGAGGGACAGCTGGTTT	AACAAGTACCCCACGCCATA
Tnnt2_T3	CCCAAGTCGCAGTGAGTTCT	ACGTGATTGACAGGACCACC
Tnnt2_T4	CCTGCCTCAGTTTCAGTGCT	GTGGCCACATGACTCAGGAC
Myh6_M1	CACCGTAGCCTATGGCTCTC	CGGCACTCTTAGCAAACCTC
Myh6_M2	CTGGCTCCCCAGAGACAAAG	GCAGCTGTTCAGTTCTGTGC
Myh6_M3	CAGCTGAGCCTATGACCCAC	TGAGCAAGCACTAACCCTCC

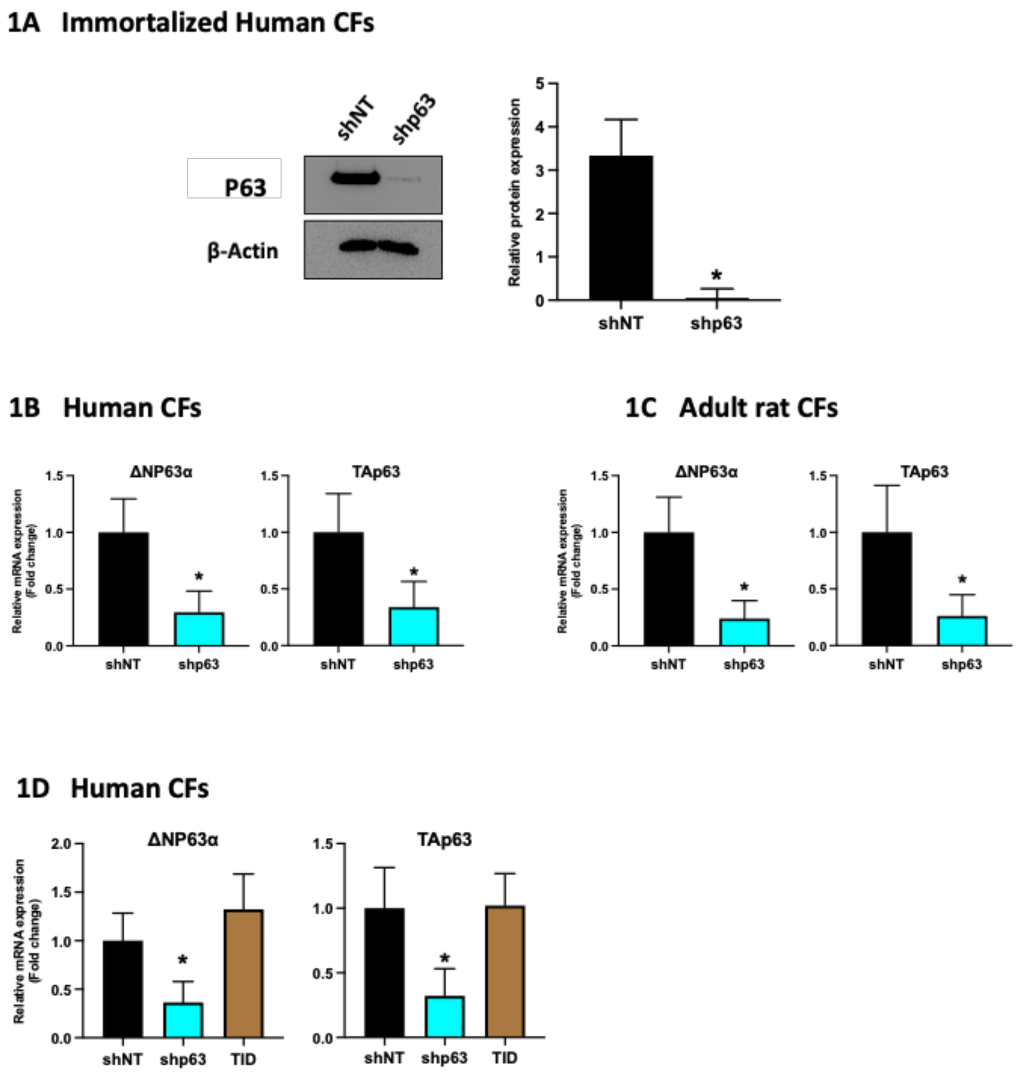

Figure S1. *p63* shRNA validation. **A)** Western analysis of P63 protein levels in immortalized human cardiac fibroblasts transfected with lentiviruses encoding shNT (non-targeting) and *p63* shRNA after 10 days (Supplemental Figure S6). Right panel shows quantified relative abundance (n = 3; *p < 0.001 versus shNT). **B, C,** qRT-PCR analysis of primary human and rat cardiac fibroblasts, respectively, demonstrating decreased expression of *p63* isoforms (ΔNp63α, TAp63α) 2 weeks after lentiviral administration as depicted in the figure. (n = 3; *p < 0.001 versus shNT). **D)** *p63* gene expression levels were assessed via qRT-PCR 2 weeks after p63-TID administration. (n = 3; *p < 0.001 versus shNT).

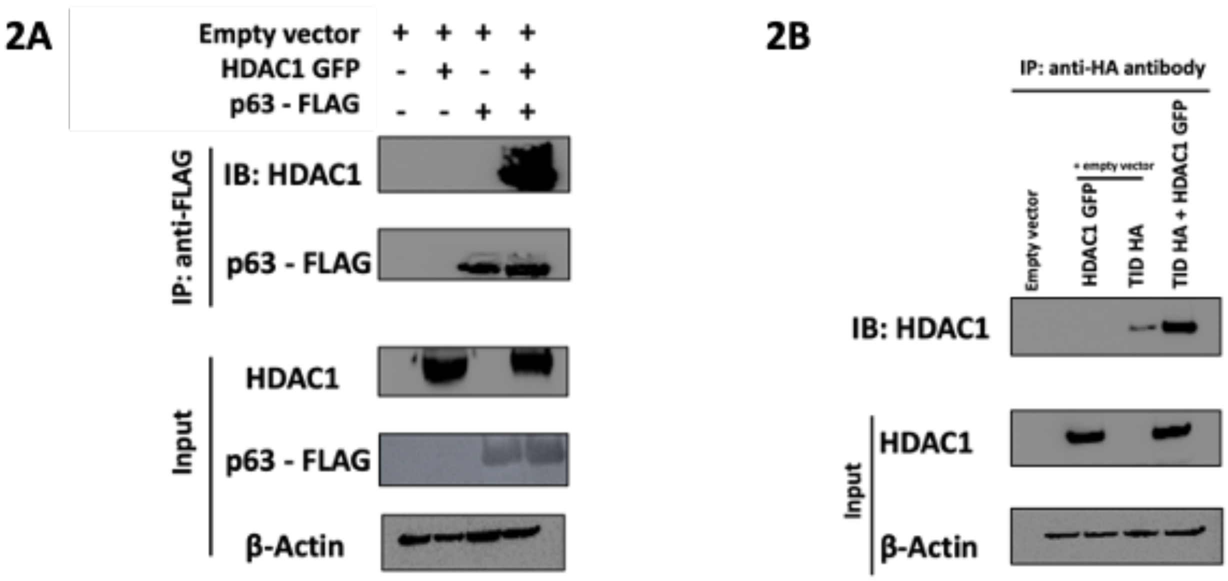

Figure S2. p63 interacts with HDAC1 and p63-TID is required for their interaction. **A)** 293T cells were transfected with HDAC1 and/or p63–FLAG vectors and with empty vector (pcDNA3.1 control) and subjected to Co-IP analysis with anti-FLAG antibody. Beta-actin was used as loading control. IB, immunoblot; IP, immunoprecipitation. **B)** TID-HA and HDAC1 vectors were co-expressed in 293T cells with or without empty vector (pcDNA3.1 control) followed by Co-IP analysis with anti-HA antibody. Beta-actin was used as loading control. IB, immunoblot; IP, immunoprecipitation (Supplemental Figure S7).

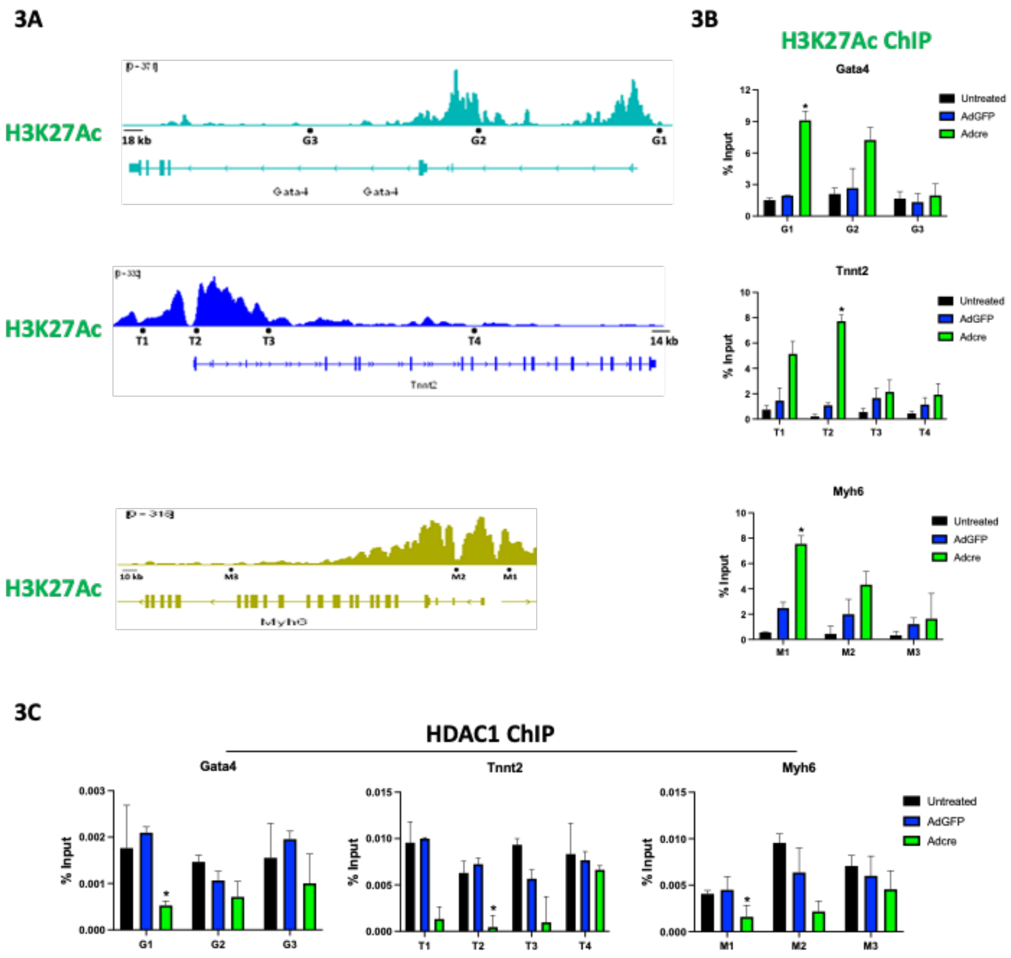

Figure S3. p63 recruits HDAC1 at cardiogenic gene promoter regions. A) H3K27Ac relative binding peaks at indicated cardiac gene loci from published ChIP-seq data (ENCSR675HDX). The gene name initial followed by numbers represent a series of amplicons which correspond to the peaks. ChIP-qPCR of **B, C)** H3K27Ac and HDAC1 at the indicated cardiogenic gene loci on untreated, AdGFP or AdCre (*p63*$^{-/-}$) treated *p63* flox/flox MEFs at day 7. The intergenic region on chromosome 12 (chr12) and the β-actin encoding Actb were used as negative and positive control (data not shown). ($n = 3$; *$p < 0.01$ versus AdGFP).

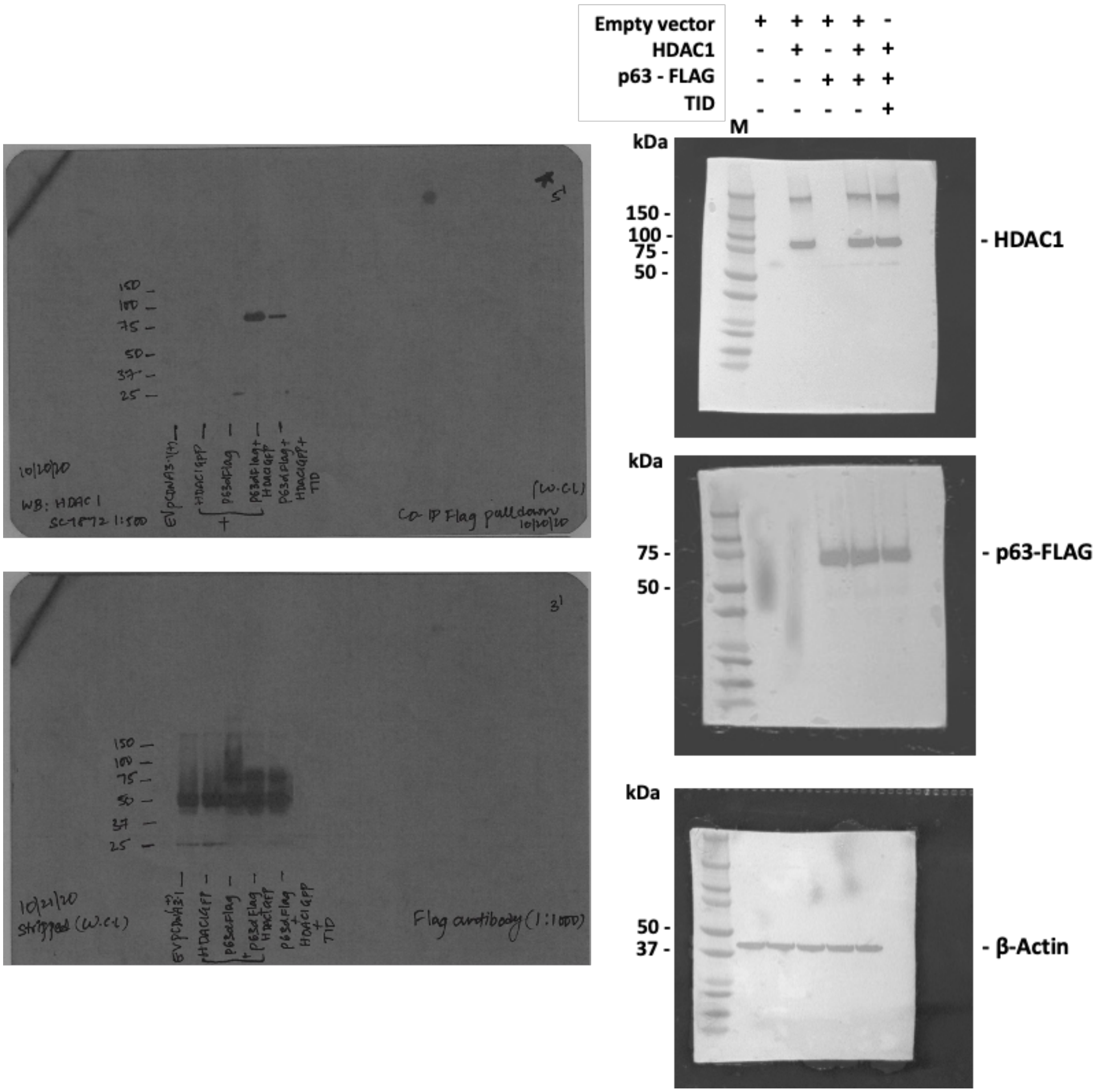

Figure S4. Western blot raw images, Figure 4A. FLAG co-immunoprecipitation assay in 293T cells transfected with HDAC1, p63-FLAG and/or p63-TID vectors, showing TID interference in p63-HDAC1 binding. Beta-actin was used as loading control.

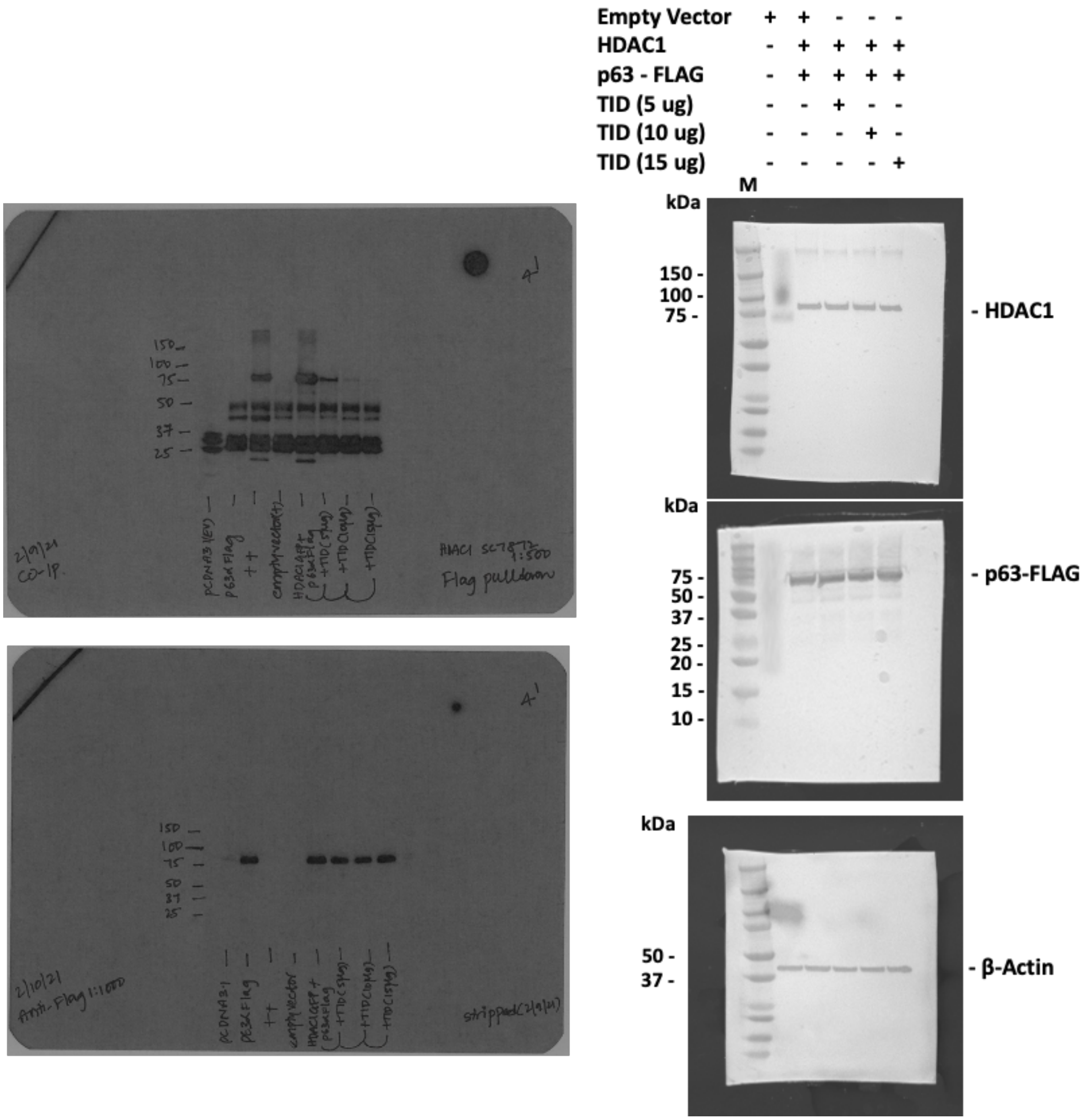

Figure S5. Western blot raw images, Figure 5A. FLAG co-immunoprecipitation assay in 293T cells transfected with HDAC1, p63-FLAG and/or p63-TID vectors at three different p63-TID dosages showing increasing interference in p63-HDAC1 binding as a function of p63-TID dosage. Beta-actin was used as loading control.

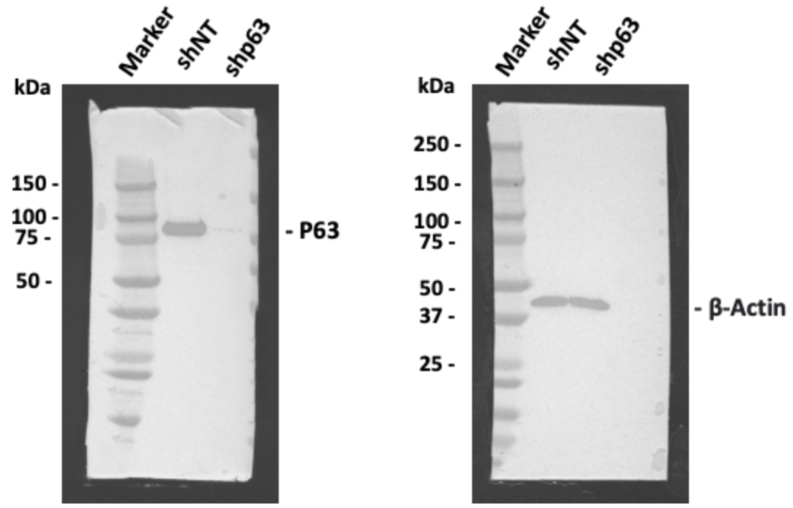

Figure S6. Western blot raw images, Supplemental Figure S1. A) *p63* shRNA validation. Western analysis of P63 protein levels in immortalized human cardiac fibroblasts transfected with lentiviruses encoding shNT (non-targeting) and *p63* shRNA after 10 days.

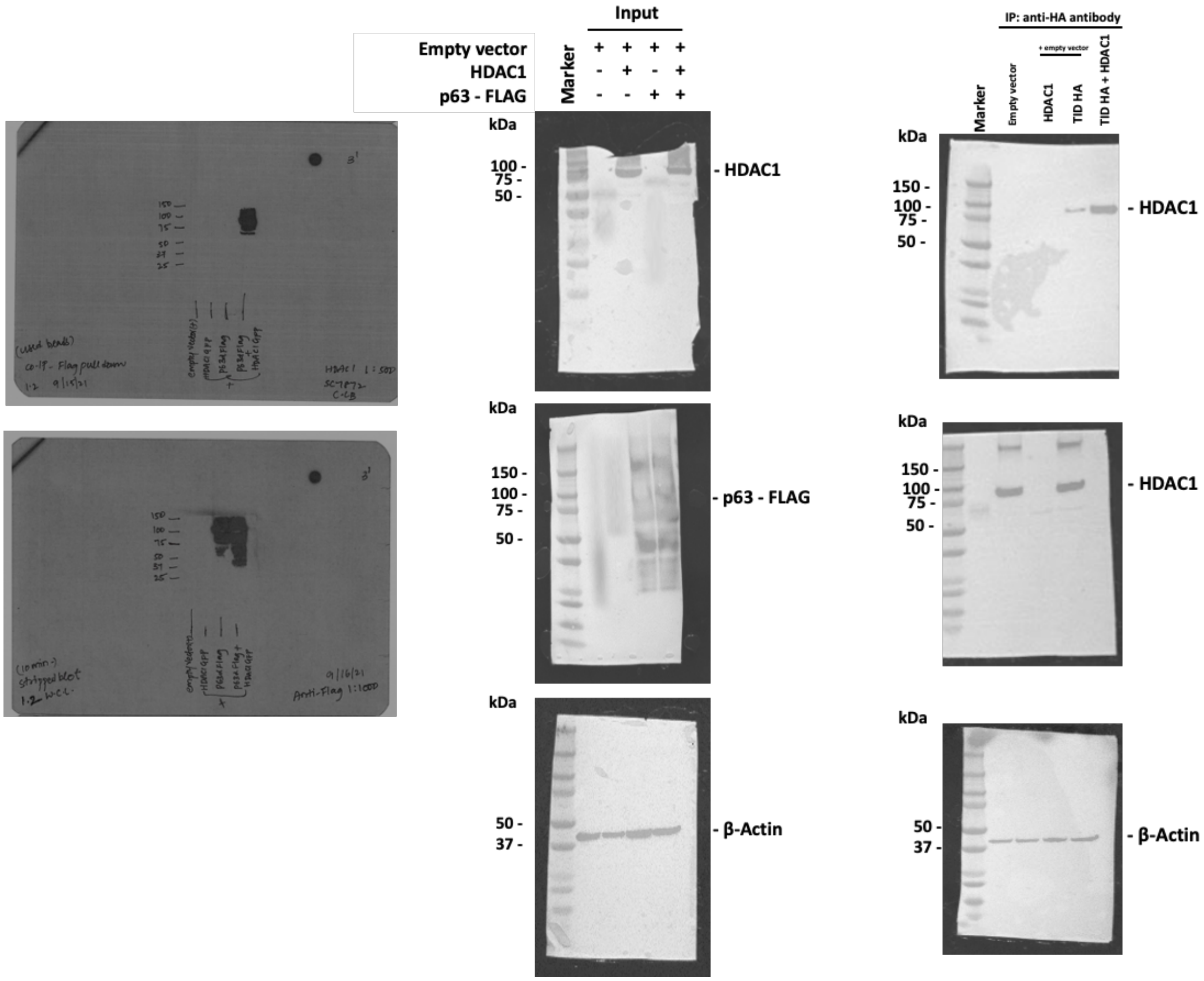

Figure S7. Western blot raw images, Supplemental Figure S2. p63 interacts with HDAC1 and p63-TID is required for their interaction. **A)** 293T cells were transfected with HDAC1 and/or p63–FLAG vectors and with empty vector (pcDNA3.1 control) and subjected to Co-IP analysis with anti-FLAG antibody. Beta-actin was used as loading control. IB, immunoblot; IP, immunoprecipitation. **B)** TID-HA and HDAC1 vectors were co-expressed in 293T cells with or without empty vector (pcDNA3.1 control) followed by Co-IP analysis with anti-HA antibody. Beta-actin was used as loading control. IB, immunoblot; IP, immunoprecipitation

Supplemental Video Legends: p63 silencing induces iCM contractility; Videos S1, S2, and S3.

Video S1. Video showing that human cardiac fibroblasts treated with GMT did not demonstrate contractions. Cells treated with GMT + shp63 also did not demonstrate contractions (video not shown).

Video S2. Video showing that human cardiac fibroblasts treated with shp63 + H/M vectors contracted synchronously with surrounding rat cardiomyocytes.

Video S3. Video showing that human cardiac fibroblasts treated with p63-TID + H/M vectors contracted synchronously with surrounding rat cardiomyocytes.
